# Supplementary material for: Walk-in mental health: Bridging barriers in a pandemic
Source: PLoS One. 2024 May 31;19(5):e0302543. doi: 10.1371/journal.pone.0302543 (PMC11142450; doi:10.1371/journal.pone.0302543)
Supplement: Attachment — Submitted filename: Response Letter to Reviewers.docx [file pone.0302543.s001.docx]

Response to Reviewers

Dr. Yaara Zisman-Ilani

Academic Editor

PLOS ONE

Submission ID: PONE-D-23-00898

Dear Dr. Zisman-Ilani and Reviewers,

We appreciate you and the reviewers for your precious time in reviewing our paper and providing valuable comments. These fantastic and insightful comments have led to possible improvements in the current version.

The authors have carefully considered the comments and tried our best to address every one of them. We hope the manuscript after careful revisions meets your standards. The authors welcome further constructive comments if any.

Below we provide the point-by-point responses. All modifications in the manuscript have been highlighted in red.

Sincerely,

Ian Wellspring (M.A.), Ph.D. Student in Clinical Psychology

Department of Psychology

University of British Columbia (Okanagan)

Email: [iwellspr@mail.ubc.ca](mailto:iwellspr@mail.ubc.ca)

Kirthana Ganesh (M.Sc.), Ph.D. Student in Clinical Psychology

Department of Psychology

University of British Columbia (Okanagan)

Email: kirthana.ganesh@ubc.ca

Dr. Kimberly Kreklewetz (Ph.D.), Lecturer

Department of Psychology

University of British Columbia (Okanagan)

Email: kimberly.kreklewetz@ubc.ca

Journal Requirements

When submitting your revision, we need you to address these additional requirements.

1. Please ensure that your manuscript meets PLOS ONE's style requirements, including those for file naming. The PLOS ONE style templates can be found at

https://journals.plos.org/plosone/s/file?id=wjVg/PLOSOne_formatting_sample_main_body.pdf and

<https://journals.plos.org/plosone/s/file?id=ba62/PLOSOne_formatting_sample_title_authors_affiliations.pdf>

**Response:** Thank-you very much and we have updated the formatting.

2. Thank you for stating in your financial disclosure: "The Walk-in Wellness clinic at the University of British Columbia (Okanagan) is funded by the private donors who wish to remain anonymous. The funders had no role in study design, data collection and analysis, decision to publish, or preparation of the manuscript." PLOS ONE requires you to include in your manuscript further information about the funder so that any relevant competing interests can be assessed. Please respond to the following questions:

a. Please state whether any of the research costs or authors' salaries were funded, in whole or in part, by a tobacco company (our policy on tobacco funding is at <http://journals.plos.org/plosone/s/disclosure-of-funding-sources>)

b. Please state whether the donor has any competing interests in relation to this work (see http://journals.plos.org/plosone/s/competing-interests) .

c. Please state whether the identity of the donor might be considered relevant to editors or reviewers’ assessment of the validity of the work.

d. If the donors have no perceived or actual competing interests, please state: “The authors are not aware of any competing interests”.

This information should be included in your cover letter. We will amend your financial disclosure and competing interests on your behalf.

**Response:** Thank-you very much and we have updated the information. Neither the research costs nor authors salaries were funded by a tobacco company. The donor does not have any competing interest, and as such the identity would not be considered relevant to the editors or reviewers. The authors are not aware of any competing interests.

3. Thank you for stating the following in the Acknowledgments Section of your manuscript:

"The Walk-in Wellness clinic at the University of British Columbia (Okanagan) is funded by the private donors who wish to remain anonymous. " We note that you have provided funding information that is not currently declared in your Funding Statement. However, funding information should not appear in the Acknowledgments section or other areas of your manuscript. We will only publish funding information present in the Funding Statement section of the online submission form. Please remove any funding-related text from the manuscript and let us know how you would like to update your Funding Statement. Currently, your Funding Statement reads as follows: "The Walk-in Wellness clinic at the University of British Columbia (Okanagan) is funded by the private donors who wish to remain anonymous. The funders had no role in study design, data collection and analysis, decision to publish, or preparation of the manuscript. " Please include your amended statements within your cover letter; we will change the online submission form on your behalf.

**Response:** Thank-you very much. We have removed the funding information from the acknowledgement section. The funding statement can remain as it is stated above given that there are no competing interests that we are aware of, nor the funding provided is from a tobacco company or go directly towards the research salaries.

4. In your Data Availability statement, you have not specified where the minimal data set underlying the results described in your manuscript can be found. PLOS defines a study's minimal data set as the underlying data used to reach the conclusions drawn in the manuscript and any additional data required to replicate the reported study findings in their entirety. All PLOS journals require that the minimal data set be made fully available. For more information about our data policy, please see http://journals.plos.org/plosone/s/data-availability.

""Upon re-submitting your revised manuscript, please upload your study’s minimal underlying data set as either Supporting Information files or to a stable, public repository and include the relevant URLs, DOIs, or accession numbers within your revised cover letter. For a list of acceptable repositories, please see http://journals.plos.org/plosone/s/data-availability#loc-recommended-repositories. Any potentially identifying patient information must be fully anonymized.

Important: If there are ethical or legal restrictions to sharing your data publicly, please explain these restrictions in detail. Please see our guidelines for more information on what we consider unacceptable restrictions to publicly sharing data: http://journals.plos.org/plosone/s/data-availability#loc-unacceptable-data-access-restrictions. Note that it is not acceptable for the authors to be the sole named individuals responsible for ensuring data access.

We will update your Data Availability statement to reflect the information you provide in your cover letter.

**Response:** Thank-you very much for highlighting this information. However, the current data would not be able to be publicly shared for two primary reasons. Firstly, the CAT-MH data is from a third-party service and as such the current authors do not have legal authority to be able to share the data. The data is owned by the developers of the CAT-MH: Adaptive Testing Technologies. In order to access the data, a researcher would need to contact the primary research (Ian Wellspring) who would then need to coordinate with representatives from CAT-MH, as well as directors of the Walk-in Service to ensure that all data sharing legal requirements are being adhered to by both CAT-MH and the host institution (UBCO). Secondly, client feedback data is sensitive data given that some of the information provided by clients directly mentions their client concerns, what was discussed during their confidential session, and/or includes information that would remove their anonymity. In particular, and as mentioned in the manuscript, patient confidentiality is a primary consideration and as such that data would not be available to be publicly shared.

5. Please include your full ethics statement in the ‘Methods’ section of your manuscript file. In your statement, please include the full name of the IRB or ethics committee who approved or waived your study, as well as whether or not you obtained informed written or verbal consent. If consent was waived for your study, please include this information in your statement as well.

**Response:** Thank-you very much and this has been included in the “Procedure” section.

Reviewers' comments

Reviewer #1: Thank you for the opportunity to review this manuscript that addresses the topic of COVID-19 effects on mental health, which is still a pressing issue that requires more understanding and addressing. The efficacy of IPC/WIWC is also interesting and can offer a solution to existing gaps in mental health services. The mixed-methods approach provides a broad view of clients' experiences. However, I find that the paper presents two completely different subjects (1. Mental health during and before COVID-19; 2. Clients' satisfaction with WIWC/IPC), and although the authors try to link them in the discussion, they are presented as separate through most of the manuscript and also in the study's procedure (since the feedback forms are not dated, COVID-19 associations with clients' satisfaction were not explored). Therefore, I think the authors should consider removing the parts about clients' feedback regarding WIWC (perhaps presenting them in a different paper), and focusing only on mental health before and during COVID-19. This was the most pressing concern, and it caused confusion throughout the manuscript, from the title to the discussion. Below I provided more thoughts and feedback, which I hope will be of help.

Title:

1. The title of the paper should be reconsidered. After reading it, I expected that the focus would be mental health during the pandemic. I didn't expect such a large volume about IPC itself (especially in the introduction). I recommend rethinking the title so that it better reflects the content of the paper, or as I suggested above, reorganizing the paper so it includes only one of the subjects.

**Response:** Thank-you for the insight! The title of the manuscript has been adjusted to better reflect the study, and the manuscript itself has been re-organized.

Abstract:

2. The study aims should be included before describing the CAT-MH.

**Response:** Thank-you and this has been updated.

3. The sentence "Profile analysis indicated a significant difference in mental health profiles" should be made clearer and more accurate to the results it describes. It is the first sentence to describe the main result, so it should be clear and simple.

**Response:** Thank-you and this has been updated to state “Profile analysis of the CAT-MH data indicated that clients had higher rates of depression before COVID-19 (*M* = 64.2, *SD* = 13.07) as compared to during the pandemic (*M* = 59.78, *SD* = 16.87).”

4. The paper reports a surprising finding: lower depression scores and higher suicidality flags. It should be acknowledged and explained (in the discussion, but also in the abstract) that these two findings together are unexpected.

**Response:** Thank-you and this has been updated to state “The lower reported rates of depression but higher rate of suicidality during the pandemic was an unanticipated finding that contradicted prior research, to which possible explanations are explored.” Given word count restrictions, a precise explanation is not provided in the abstract but it is elaborated upon in the discussion.

5. Please remove the word "the" in "the private donors" under financial disclosure.

**Response:** Thank-you and this has been updated.

6. In the last sentence, please use the full term "Integrated Primary Care (IPC)" as it is the first time this abbreviation is mentioned.

**Response:** Thank-you and this has been updated and/or removed.

Introduction:

7. In general, there is a lot of information here about IPC and not enough about mental health during the pandemic. The title and abstract focused on mental health during COVID-19 and then the introduction only describes it in one paragraph after discussing IPC for two pages. This is confusing.

**Response:** Thank-you and this has been updated. Specifically, information related to IPC has been significantly redacted, while information related to COVID-19 (and pandemics/natural disasters more broadly) has been expanded.

8. In line 112, I suggest replacing "endorsing" with "reporting".

**Response:** Thank-you and this has been updated.

9. In lines 119-121, I think you should mention that therapists' transition to videoconferencing therapy is usually due to government restrictions during the pandemic.

**Response:** Thank-you and this has been updated.

10. Lines 124-152 (describing the CAT-MH) belong in the METHODS>>MEASURES section.

**Response:** Thank-you and this information has been moved.

11. Similar to the previous comment, lines 153-164 (describing the WIWC) belong in the METHODS>>PARTICIPANTS, especially since most of the content already appears in the PARTICIPANTS section.

**Response:** Thank-you and this information has been moved and updated.

12. I found it confusing that after such a long description of IPCs, the study took place in WIWC, which lacks the "bridge" element between physical and mental health care. Therefore, I suggest removing or narrowing the part describing the benefits of IPC by highlighting the collaboration between the physician and mental health provider and the reduction of stigma.

**Response:** Thank-you and as mentioned above, information related to IPC has been decreased.

13. There is a gap between the OBJECTIVES & HYPOTHESES section which focuses on persons' mental health during COVID-19, and the INTRODUCTION which focuses on the efficacy of IPC. To better reflect the objectives, I suggest again narrowing considerably the IPC part in the introduction and representing more fully the effects of the pandemic on mental health (for example, citing studies not just from Canada, adding literature about mental health during previous pandemics). In general, I recommend clarifying what is already known, what the knowledge gaps are and then using this to justify the study rationale.

**Response:** Thank-you and as mentioned above, information related to IPC has been decreased. Further, information has been included discussing mental health during other pandemics/natural disasters and there has been a re-focus on single session therapy.

14. I don't understand how the two objectives are related. It seems to me that these are two completely different subjects: 1) mental health during COVID-19. 2) Clients' satisfaction with the WIWC. Because the feedback forms are without dates and thus don't allow for a comparison between before and during the pandemic, I think it will be better to remove the second objective and focus this paper only on mental health during the pandemic.

**Response:** Thank-you and we have attempted to provide a clearer connection between the two study objectives in the manuscript.

Methods:

15. When describing participants and the WIWC, please elaborate more about the nature of the intervention offered. For example, what kind of professionals work there, how many sessions are offered, what kind of therapeutic approaches are used?

**Response:** Thank-you and we have included more specific information relate the professionals in the service, number and extent of sessions, as well as examples of typically utilized tools and resources.

16. Please specify the dates of data collection.

**Response:** Thank-you and it is stated in the manuscript that data for before the pandemic spans November 2018 to March 11, 2020, while during the pandemic is from March 11, 2020 to July 2022.

17. Please clarify does each provider read the patient's CAT-MH results before the session? Namely, is the CAT-MH used so that providers will know which issues to focus on during the current session?

**Response:** Thank-you and we have add clarification to note that the CAT-MH is reviewed by service providers prior to starting their session.

18. In line 242, the authors use an example of coding overall severity scores. Why does the researcher need to code severity if "the CAT-MH produces an overall severity rating" (line 234)?

19. Regarding feedback forms, for clients who attended the services more than once, how many forms were analyzed? One or more?

**Response:** Thank-you and it has been clarified that only the first feedback form was reviewed from the first time they accessed services.

Analysis:

20. The description of the form's open-ended questions (lines 269-274) belongs in the MEASURES section. There is a need for clarification because in the MEASURES>>FEEDBACK FORM section the authors write that the form includes one open-ended question and three multiple-choice questions. Then in the ANALYSIS PLAN section the authors write that the form includes other open-ended questions.

**Response:** Thank-you and there has been clarification added to note what questions are asked in the feedback form, and the information has been moved to the measures section.

21. The authors report having 693 records and 216 feedback forms of these records being analyzed. How was it decided which feedback forms to analyze?

**Response:** Thank-you and there has been clarification added to note that the feedback forms were optional for clients to complete, and indeed were not as readily completed as compared to the CAT-MH. As such, all available feedback forms that were completed were coded.

Results:

22. Again, I think the whole part about treatment satisfaction should be removed (and perhaps reported in a separate paper). However, if you stick to it as is, please make sure that throughout the paper the two subjects appear in the same order. For example, you start the introduction and objectives with the COVID-19 issue, but you start the results with the satisfaction issue. This is confusing.

**Response:** Thank-you and there has been clarification added to the paper (as discussed above), and the organization has been made consistent across the paper.

23. In line 292, satisfaction with what? Treatment? Please specify.

**Response:** Thank-you and there has been clarification added to note that it is satisfaction with treatment.

24. How do you explain the gap between suicidality scores presented in the table (where during COVID the score is a little lower than the score before Covid), to the difference in suicidality flags according to chi-square?

**Response:** Thank-you for highlighting this difference. However, as reported in the table the difference in the suicidality scores is non-significant (*p* = .62). Further, in terms of practical significance, this is only a difference in severity scores of 0.38. As such, there is not an interpretation provided given the minimal and non-significant difference in scores. However, it is acknowledged that suicidality scores remained consistent while suicidality flags increased. An additional interpretation has been included in the paper to discuss this.

25. I suggest presenting the results not according to quantitative/qualitative, but according to the two study objectives.

**Response:** Thank-you and we re-organized and adjusted the headings to reflect study objectives.

26. In line 391, I suggest deleting "Fig 2 highlights that" and instead starting the sentence with "Clients were appreciative…".

**Response:** Thank-you and this has been updated.

27. In INFORMATIONAL SUPPORT, it would help if the reader knows what kind of techniques/plans were offered by providers. Otherwise, it sounds vague. This relates to comment no. 15.

**Response:** Thank-you and we have provided increased clarification about types of information and resources provided to clients.

Discussion:

28. The sub-titles should be broader. For example, instead of CAT-MH >>> MENTAL HEALTH BEFORE AND DURING COVID-19.

**Response:** Thank-you and the headers have been updated accordingly.

29. The inconsistency between the results of this study and other studies regarding depression is not well explained.

**Response:** Thank-you and have elaborated and provided increased clarification for these results.

30. In lines 423, 425, please delete the word "for" in "for before the COVID-19…".

**Response:** Thank-you and this has been updated.

31. In line 440, the sentence "as well as how the questions are phrased on the CAT-MH" belongs in the next paragraph and not here.

**Response:** Thank-you and this has been updated.

32. The explanation regarding creativity seems far-fetched since the study didn't ask about clients' creative activities during or before the pandemic.

**Response:** Thank-you and this information has been removed from the paper.

33. Please rephrase lines 459-462. It is not clear enough. For instance, in line 461, "higher" than what?

**Response:** Thank-you and this has been updated.

34. In line 482, what do you mean by "reduced responsibility"? Lines 482-487 don't seem to be related to the subject described in this section.

**Response:** Thank-you and this has been clarified.

35. The possibility that people may feel more comfortable disclosing suicide ideation via videoconference is interesting. However, the authors didn't address the dissonance between the two findings reported here (lower depression scores and higher suicidality flags). This should be addressed and attempted to be explained.

**Response:** Thank-you and increased clarification has been provided about the discrepancy has been discussed.

36. In CLIENT FEEDBACK (which also should be replaced with a broader title), the first sentence should be removed.

**Response:** Thank-you and this has been updated.

37. In line 493, the sentence starting with "While no single…" is not clear.

**Response:** Thank-you and this has been updated.

38. In lines 505-506, the authors write "…directly influenced their opinions of IPC". The cited paper reports an association, not a causal relationship. Please rephrase.

**Response:** Thank-you and this has been updated.

39. Under LIMITATIONS, when the authors write about the lack of demographic data, I suggest addressing also the difficulty of generalizing or reaching general conclusions.

**Response:** Thank-you and statements have been included highlighting the limited generalizability.

40. In lines 534-535, I think the PAI and MMPI are not relevant here, as they are both very long and time-consuming.

**Response:** Thank-you and this section has been removed.

41. Can you highlight the added contribution of this study to the research literature?

**Response:** Thank-you and there has been clarification added in the discussion section about the unique contributions of this study.

42. Are there specific recommendations arising from these data?

**Response:** Thank-you and there has been specific recommendations included in the conclusion section of this study.

Conclusions:

43. In line 562, please change "within" to "among".

**Response:** Thank-you and this has been updated.

44. I think this section needs more work. Specifically, the two subjects of the study (mental health during and before covid and satisfaction from WIWC) are intertwined here and should be separated. For example, in lines 574-576 the authors write about the effectiveness of services "both before and during COVID-19". However, since the feedback forms were not dated, referring to before and during COVID here is irrelevant.

**Response:** Thank-you and we have added increased clarification in this section, as well as more clearly delineating the two objective areas.

References:

45. References no. 77,78 are the same reference.

**Response:** Thank-you and this has been addressed.

Reviewer #2: The authors present findings from Computerized Adaptive Testing -- Mental Health (CAT-MH) at intake of a outpatient mental health clinic as well as thematically coded findings from open-ended questions and 3 scaled satisfaction questions completed after their (seemingly first, but this is not obvious from description) session both prior to onset of COVID and during COVID (November 2018 to March 11, 2020 and March 11, 2020, to June 2022, respectively). They describe two objections: 1) "to utilize CAT-MH data to measure the nature and severity of mental health concerns of clients who accessed a walk-in mental health clinic before and during the COVID-19 pandemic" and 2) to explore clients experiences with the walk in clinic through qualitative analysis of the open-ended questions (and given what they report, apparently also quantitative analysis of the 1-100 scales).

Regarding (1), they hypothesized that that depression, anxiety, suicidality, and substance use subscales of the CAT-MH would be worse for those presenting after COVID. In contrast, they present the interesting finding that the only means on subscales of the CAT-MH for the pre-COVID group and the during COVID group were not significantly different, with the exception of the depression subscale, where the pre-COVID group scores reflected significantly greater depression subscales.

Regarding (2), they present findings that the mean score (1-100) for finding the service helpful was 83.33, that for finding the service to address the clients concerns was 82.47, and that for overall satisfaction was 88.22--all reflecting high client satisfaction. They also present thematic findings on open-ended questions that clients found the service to be emotionally supportive; that the interventions to include helpful components (which they label "informational support," but for which many themes do not obviously have to do with providing particular types of information [e.g., that this "was a great way to 'break the ice'"); that clients identified "logistical considerations that appear to reflect reduction in barriers to accessing care' and that concerned limitations of the service (e.g., that it was too brief in length).

The authors conclude that "taken together, the results demonstrate the effectiveness and efficacy of services modeled on IPC in reducing barriers to accessing mental health care, both before and during the COVID-19 pandemic. The results further illustrate that IPC is a viable service delivery model to mitigate the challenges experienced by the healthcare system that merits further investigation and consideration. There are other important and integral research areas to consider, including the long-term effectiveness of IPC [i.e., Integrated Primary Care]."

The finding regarding depression is interesting and the exploratory findings are suggestive. However, the paper suffers from several flaws:

1) The intervention is not IPC. The authors briefly note this on p. 8, lines 188-192. There they write: "It should be noted that the WIWC is modeled on the principles of IPC, but it in and of itself is not an integrated primary care clinic as only mental health professionals provide services. However, the WIWC has fostered various relationships within the Okanagan community and actively collaborates with other service providers to ensure ease of services." The authors do not provide a detailed description of the nature of the walk-in clinic and certainly do not operationalize its model robustly. They do note that services were virtual during the pandemic and they do describe the clinic model as follows: "short-term solution-focused mental health support is offered to individuals who are often referred by family physicians, walk-in medical clinics, or other community organizations, despite an absence of colocated care."

However, this does not appear sufficient to warrant the claim that this is sufficiently like IPC or any adaptation of IPC for their findings to be informative about IPC. They note that sessions are 30 minutes in discussion of the thematic analysis, and they also note in that section that typical services are 3-4 sessions. The rationale for this is unclear. Regardless, with a lack of primary care services, it is unclear that it has any relation to integrated care except perhaps (1) trying to expand access, (2) offering brief interventions, and (3) getting referred by primary care. Importantly, an internet search suggests that the clinic is staffed by graduate psychology students, supervised by psychology faculty. It is unclear what role for psychopharmacology is present, and this would appear to be a major difference with many forms of IPC as the primary care physicians (and on some models psychiatric professionals) can offer psychopharmacological interventions. The parameters and criteria for the interventions are also not adequately detailed to assess their claims about efficacy/effectiveness in the conclusion. It would be helpful to know more about what criteria are used for discharge, completion, or referral out to higher levels of care. It would also be helpful to have a discussion comparing their criteria more fully to fidelity IPC models if the authors insist on this comparison.

**Response:** Thank-you for the insightful comments and feedback. We have provided increased clarification about the nature of the walk-in services and attempted to operationalize it more clearly in the methods section of the paper. We have also highlighted the information about referring clients to more appropriate services based on the presenting concern of the client. In general, we have decreased the emphasis on IPC given the disconnect and provided increased clarification and background information related to single-session therapy (which is utilized in IPC) that more closely adheres to the walk-in services. We’re more than open to discussing this in greater detail.

2) It is unclear why these two objectives relate to each other in a meaningful way for a single paper. Epidemiological considerations during COVID and the "effectiveness and efficacy" of IPC (on which see below) are quite different topics. Neither objective has a robust additional data with which to explore the various explanations that the authors suggest may explain their findings. This may be understandable given limitations of their non-identifiable dataset, but it raises the sense that two different topics don't have any obvious relation. Moreover, there may be some secondary analyses that might be possible with their data. For instance, is there variation in severity of CAT-MH scores across time during COVID or do rates o presentation/referral/intake vary during COVID. It is not clear that the period between March 11, 2020 and June 2022 should be taken as one homogenous mental health period. These findings would be more informative in order to appropriately interpret their CAT-MH findings.

Given that the topics appear different and if additional exploratory analyses are not possible with whatever other data the authors may have on outcomes, the authors might consider dividing this into two briefer studies submitted as brief reports independently.

**Response:** Thank-you for the insight and feedback. We provided increased clarification and adjusted the focus of the paper to more accurately reflect refined objectives and considerations. Please see the objectives, results, and discussions section for reflective changes. We understand the reviewer’s concerns regarding the consideration of the period between March 11, 2020 and June 2022 as a single time period. In the absence of clear theoretical rationale to further separate this group into smaller ones (i.e., there has been minimal societal change, COVID-19 is still considered a pandemic, etc.), we believe that from a mental health perspective, it is reasonable to consider this as a homogenous group in the present study, due to the salience of mask mandates, physical distancing, etc., that were present throughout this time.

3) The exploratory components of the study appear to be preliminary assessments for implementation outcomes. A description of whether the 1-100 point scales had greater than two anchors would be helpful. There are a number of validated implementation scales. It is acceptable to use these 1-100 points scales without validated psychometrics rather than validated implementation scales with standardized psychometrics. However, further description of the anchors of these scales (or any comparison where the scales are used elsewhere) would help.

**Response:** Thank-you and increased clarity has been provided about the anchors for this question.

4) There conclusion that "the results demonstrate the effectiveness and efficacy of services modeled on IPC in reducing barriers to accessing mental health care" appears to be overstated for two reasons. (A) The fact that this is the model is unlike IPC as noted above. (B) The exploratory findings reflect an (early phase) implementation study. There is no clinical outcome with which to assess effectiveness or efficacy as standardly understood. Even if we allow leeway in noting that the authors are speaking of "efficacy ... in reducing barriers" some robust operationalization (and likely comparison) would be needed to "demonstrate ... efficacy." There are a variety of ways in which implementation might be measured and implementation outcomes can be compared across groups, but this doesn't appear to be considered.

**Response:** Thank-you and we have rephrased the outcomes to decrease the emphasis on efficacy and effectiveness given the lack of follow-up data and clear outcome measures (i.e., pre-post measures). Information has also been included to more accurately reflect some of the key takeaways and recommendations from this study that may not be directly relate to efficacy.
