# Supplementary material for: Walk-in mental health: Bridging barriers in a pandemic
Source: PLoS One. 2024 May 31;19(5):e0302543. doi: 10.1371/journal.pone.0302543 (PMC11142450; doi:10.1371/journal.pone.0302543)
Supplement: Attachment — Submitted filename: Response Letter to Reviewers.docx [file pone.0302543.s002.docx]

Response to Reviewers

Dr. Yaara Zisman-Ilani

Academic Editor

PLOS ONE

Submission ID: PONE-D-23-00898

Dear Dr. Zisman-Ilani and Reviewers,

We appreciate you and the reviewers for your precious time in reviewing our paper and providing valuable comments. These fantastic and insightful comments have led to possible improvements in the current version.

The authors have carefully considered the comments and tried our best to address every one of them. We hope the manuscript after careful revisions meets your standards. The authors welcome further constructive comments if any.

Below we provide the point-by-point responses. Tracked changes have been utilized to highlight the updates and revisions.

Sincerely,

Ian Wellspring (M.A.), Ph.D. Candidate in Clinical Psychology

Department of Psychology

University of British Columbia (Okanagan)

Email: [iwellspr@mail.ubc.ca](mailto:iwellspr@mail.ubc.ca)

Kirthana Ganesh (M.Sc.), Ph.D. Candidate in Clinical Psychology

Department of Psychology

University of British Columbia (Okanagan)

Email: kirthana.ganesh@ubc.ca

Dr. Kimberly Kreklewetz (Ph.D.), Lecturer

Department of Psychology

University of British Columbia (Okanagan)

Email: kimberly.kreklewetz@ubc.ca

Reviewers' comments

Reviewer #1: I appreciate the authors' work in response to the comments. In particular, the elaboration of the pandemic's impact on mental health in the introduction and the explanations of the outcomes regarding depression and suicidality are now much more comprehensive. However, I still think the link between the two subjects (service satisfaction and mental health before and during COVID) is not strong and doesn't justify including them in the same paper. Below I provide some more specific comments.

Title and Abstract
1. The revisions made in the title and abstract highly improved these sections.

Thank-you for the support and feedback.

Introduction
2. In line 68, the word "however" should be removed because this sentence doesn't seem to contradict the previous sentence.

This has been amended.

3. Lines 100-103 belong in the METHODS section, not the introduction.

This has been amended.

4. I still don't understand why the authors describe the service as IPC. Since the service in the current study doesn't include physicians or any kind of multidisciplinary team, I don't see why it is considered IPC. Therefore, I think the paragraphs about IPC (lines 104-126) should be removed.

This has been amended and increased clarification has been provided.

5. Lines 119-125 seem irrelevant to the paper's topic.

This has been amended.

6. I think there is still too much information about SST and IPC and the introduction is still highly unbalanced, with two and a half pages about SST/IPC and only one page about COVID-19. It is also confusing that the authors write about SST, IPC, walk-in clinic, and CC. The part about CC (line 109) can be removed from the introduction because it is described again in the PARTICIPANTS section.

The mentioned comments have been addressed and the introduction has been updated to more accurately reflect a balanced perspective on SST and COVID-19 related mental health concerns.

7. The introduction of COVID-19 (line 127) is not well connected to the IPC paragraph that precedes it.

This has been amended.

8. Personally, I think it would be better if Covid-19 is described first and SST is described afterward, with lines 149-151 serving as the bridge between these two subjects.

This has been amended.

Objectives
9. It is unclear how lines 159-163 explain what follows them (the use of the CAT-MH).

This has been amended.

Measures
10. Lines 192-196 belong in the CAT-MH paragraph (lines 218-257).

This has been amended.

11. The CAT-MH section is a bit long. I suggest narrowing it down.

The CAT-MH description has been condensed and re-worded for increased clarification.

12. In line 280, "feedback forms are not dated" can be deleted because it is also written in line 293.

This has been amended.

Discussion
13. Line 532, "The current study also found a higher rate of ‘positives’.." is missing "during the pandemic compared to before the pandemic".

This has been amended.

14. In line 547, "The present study also illustrated that.." should be rephrased because this is not an additional finding but an explanation of the two findings described earlier.

This has been amended.

15. The explanation in lines 559-566 is not clear enough. If you mean that clients had more time during the pandemic to seek treatment for their suicidal thoughts, then why only suicidal ideation was higher and not other symptoms as well?

There has been increased clarification provided.

Reviewer #2: The authors have improved the paper with regard to the concerns that both I and the other reviewer raised. However, I think that issues remain.

Major Concern:

1) Both reviewers were initially concerned that the manuscript was addressing to topics that were not obviously related. The authors revision has imporved this, but the rationale for the second objective given the first objective is still not as clear as it could be.

The authors state that the primary objective is exploratory analysis of qualitative feedback from clients about their experience of SST and their perceptions of its efficacy. To link this to the second objective, they write: "Further, it is acknowledged that situational and contextual factors are going to impact how services are accessed, as well as who accesses them. The role of client motivation may mean that those that sought the service may be those that were ‘ready’ for change. It is also acknowledged that those who were able to access the service were likely individuals who had privileges such as a stable internet connection and/or a working computer/tablet/cellphone. As such, a second objective of the present study was to utilize CAT-MH data to measure the nature and severity of mental health concerns of clients who accessed a walk-in mental health clinic before and during the COVID-166 19 pandemic." (Lines 159-166)

If I understand the authors correctly, this simply means something like "A secondardy objective was to contextualize the qualitative findings in regard to the nature and severity of clients' mental health concerns, as assessed by CAT-MH, because severity is known to correlate with length of time in treatment [or perhaps "time to remission" or perhaps both--here any number of citations could be given]." All the other suggestions appear speculation of potential explanations that are more appropriate in some other section (likely the discussion section). However, it's unclear if I understand them right. If I do not understand them right, the the specific claims need to be justified for the methods section.

We understand and appreciate the reviewer’s comments on this point. We have revised the study objectives section to better clarify the relationship between the two research questions. The study was primarily designed to understand the efficacy of SST as a treatment modality within a walk-in mental health clinic, and we used a mixed methods approach to comprehensively evaluate this. As COVID-19 emerged as a significant global event during this time with far-reaching mental health implications (among others) it became necessary to separate data into pre-COVID-19 and during the pandemic. We have done so with the quantitative data, but as noted, we were unable to do so with the qualitative data. As such, we have re-written portions of the section to clarify this further.

Of particular note here, their discussion of "readiness for change" and other socioeconomic determinants does not appear to be measured by the CAT-MH. Thus, if those issues are part of the objective, the study cannot in principle achieve this objective.

We understand the confusion and have removed this line to clarify.

2) While the author's revision as above gives more clarity to why two seemingly different topics are brought together into a single paper, I don't see that the authors actually "contextualize" anything in the discussion. The discussion is still offered as largely two independent topics. The secondary objective doesn't obviously contribute to contextualization of the first in the discussion. I think that if I am right about the way the authors are trying to relate the two issues, the discussion needs to reflect that this is a goal.

We have modified the text in the discussion section to clarify the relationship between the two hypotheses.

Significant Concerns to Consider Accounting For:

3) I believe the authors should consider whether they could conduct a secondary analysis for variation during the pre- and during-covid periods in (1) rate of appointments over smaller units of time and (2) severity of symptoms over smaller units of time. I understand they are conducting multiple tests and have already conducted a Bonferroni correction. While it is reasonable to set the parameters for initial analysis purposes, I believe that a secondary analysis would shed more light on the author's finding of the decreased rate of depression with concurrent the increased rate of suicidality that they saw during the Covid period. I take it the author's believe (rightly) that this is one of their most intriguing findings, but they could improve their speculations about it in the discussion through secondary analysis of how suicidality, depression, and other CAT-MH symptomology varied over time.

We understand and appreciate the reviewer’s comments on this point. We have provided graphical representations of the depression and suicide flag related data to provide increased insight. Further, we have highlighted the two months following the COVID-19 pandemic in each graph. We selected this time frame given that the WIWC services were on hold immediately following (i.e., the following 2 weeks) the COVID-19 declaration, and as such an increased timeframe was required to ensure that an accurate sample size was available. We hope that this provides increased insight into the pattern of mental health symptoms that were apparent during this time.

Two points are important here:
(a) The authors overlook several possible explanations of the apparent paradoxical finding of decreased depression with increased suicidality that variation of symptoms in time would explain. For instance, high depression scores early in Covid during and shortly after lockdown orders could be masked by increased rate of presentation that they report during Covid on the whole. It is possible that most suicdality presented at time periods that coincide with higher depression scores, but that increased rate of presentation watered down the depression score for the Covid period on the whole. If that were the case, the paradox of increased suicidality rate with decreased depression scores would merely be an artifact of the author's approach.

We appreciate the reviewer’s depth of thought on this point and have endeavored to clarify the apparent paradox in the discussion section. We also have added a graphical representation of the two most salient findings (i.e., the depression scores and suicidality flags), and have segregated them into various time frames. Our preliminary analyses showed that the changes noted in the overall data between the initial two time periods (pre and during COVID) stayed consistent. The service runs 2 days a week, and so we considered a two-month period (i.e., 16 working days). As such, we believe that the way the data has been currently divided for inferential statistics (i.e., profile analysis) is the most appropriate way to explore the research question, with robust statistical testing.

Relatedly, the authors note that they began to offer telehealth services during Covid. In my clinic (and those of my colleagues), the no show rate dropped significantly once telehealth was started. Anecdotally, that easier access seemed to have coincided with more individuals with less symptom burden presenting--even though some individuals got much sicker. (My understanding is that others observed the same phenomneon). Likewise, public attention to mental health during the covid period grew vastly, which might also have resulted in a greater presentation rate of individuals that would not otherwise have presented.

We agree that increased public attention to mental health during COVID may have played a role in an increase in individuals seeking treatment. As the clinic is a walk-in service, it is difficult to accurately compare no-shows between in-person and virtual appointments, since individuals often simply presented to the service, or called earlier in the day for a same-day appointment. Moreover, the SST model is built on the assumption that each session is stand-alone (i.e., no obligation for future appointments) and it is not a long-term therapy service in which there is an expectation that clients would attend regular appointments. No-show rates were therefore not evaluated, and are not within the purview of the modality of SST. Intuitively, it is reasonable to expect that individuals with less symptom burden may have presented during COVID due to ease of access; however, our results demonstrate that there were no significant differences in the rates of anxiety, substance use, and trauma symptoms, and the only differences were in depression and suicidality, which have explored in the discussion section.

(b) In response to my earlier suggestion they merely stated: "we believe that from a mental health perspective, it is reasonable to consider this as a homogenous group in the present study, due to the salience of mask mandates, physical distancing, etc., that were present throughout this time" in light of the fact that "there has been minimal societal change, COVID-19 is still considered a pandemic, etc." First, this hypothesis is testable by the secondary analysis above. Second, it is not clear that societal change was minimal during this time: there were multiple variations in infection rate, death rate, and hospitalization rate during these periods. There was also significant variation (at least in some locations that I'm more familiar with than the author's location) between stay at home order intensity, amount of standard public interaction, and whether or not children/parents could engage in normal school/work/recrational activities.

We agree and appreciate the reviewer’s comments to this section. We have altered the wording to this section to provide increased clarification and insight.

Minor comments:
4) Single Session Therapy is mentioned at line 77, but the acronym SST is not introduced to the next mention of SST @ line 78. The acronym should be placed after the first mention.

This has been amended.

5) The full name, Computerized Adaptive Testing - Mental Health, is used in the abstract, then CAT-MH is used in several parts of the main paper as an acronym before the full name is respicified at line 221. Please spell out the full name at first use.

This has been amended.
