# Supplementary material for: Walk-in mental health: Bridging barriers in a pandemic
Source: PLoS One. 2024 May 31;19(5):e0302543. doi: 10.1371/journal.pone.0302543 (PMC11142450; doi:10.1371/journal.pone.0302543)
Supplement: Attachment — Submitted filename: Response to Reviewers_2Apr2024.docx [file pone.0302543.s003.docx]

Response to Reviewers

Dr. Emily Lund

Academic Editor

PLOS ONE

Submission ID: PONE-D-23-00898

Dear Dr. Lund and Reviewers,

We appreciate you and the reviewers for your precious time in reviewing our paper and providing valuable comments. These fantastic and insightful comments have led to possible improvements in the current version.

The authors have carefully considered the comments and tried our best to address every one of them. We hope the manuscript after careful revisions meets your standards. The authors welcome further constructive comments if any.

Below we provide the point-by-point responses. Tracked changes have been utilized to highlight the updates and revisions.

Sincerely,

Ian Wellspring (M.A.), Ph.D. Candidate in Clinical Psychology

Department of Psychology

University of British Columbia (Okanagan)

Email: [iwellspr@mail.ubc.ca](mailto:iwellspr@mail.ubc.ca)

Kirthana Ganesh (M.Sc.), Ph.D. Candidate in Clinical Psychology

Department of Psychology

University of British Columbia (Okanagan)

Email: kirthana.ganesh@ubc.ca

Dr. Kimberly Kreklewetz (Ph.D.), Lecturer

Department of Psychology

University of British Columbia (Okanagan)

Email: [kimberly.kreklewetz@ubc.ca](mailto:kimberly.kreklewetz@ubc.ca)

Reviewers’ Comments

Reviewer #1: I appreciate the authors work on the manuscript, which is now more integrative.

Thank-you for the support and feedback.

Introduction

1. The revisions made in this section are excellent.

Thank-you for the support and feedback.

Methods

2. Line 171 “However, the number of sessions was 172 altered if extenuating circumstances were present (e.g., suicidal ideation or other forms of 173 imminent risk).” - Are there participants in the current study that attended more than 4 sessions due to such circumstances? If so it should be considered whether they should have been removed from analysis because it becomes a different kind of treatment and not necessarily SST.

We appreciate the clarification. All participants completed only one feedback form (on their first appointment). Similarly, only the CAT-MH scores from their first appointment used for the present study, as they were the most representative of the reason why clients accessed the service. This is described in lines 245-246 and 249-251 in the Procedures section of the revised document with track changes enabled. Further, an additional sentence has been added after line 171: “For the present study, only data from the client’s first appointment was collected and analyzed.”

Discussion

3. Lines 469-473: Does this sentence reflect in the results as well? Did participants address this advantage in the open ended questions?

Ease of access was a theme that emerged; however, specific references to virtual service delivery were not present. The word “likely” has been added to this sentence to communicate the extrapolation made by the researchers.

4. Lines 536-538: I suggest to remove this because it’s not directly related to the current study.

The sentence has been removed.

5. Line 606: IPC was removed from the paper so it should be removed from here also.

The term IPC has been replaced with SST.

Reviewer #2: The authors have improved the manuscript significantly. I have only minor, easily addressable comments below.

Thank-you for the support and feedback.

Substantive comments:

1. P. 33, line 559 says that a hypothesis about increased suicidality resulting from improved ease of access was "substantiated" by the qualitative findings which reflect ease of access. However, the anonymity of the authors' data precludes showing that the individuals with suicidality were the ones who reported increased ease of access. Thus, the verb should be weakened to one that doesn't suggest direct hypothesis testing occurred. "Supported" would be one alternative.

We appreciate the clarification. The line has been amended.

2. P. 35, line 606: The paper is no longer discussing IPC and hence reference to IPC needs to be replaced here.

The term IPC has been replaced with SST.

Minor grammatical comments:

1. P. 13, lines 83-84: "the Covid-19" is awkward. Perhaps either the "the" should be deleted or "pandemic" (or some similar word) should be added.

The word “the” has been deleted.

2. P. 14, line 99: consider hyphenating "anxiety-related disorders."

The term has been amended.

3. P. 14, lines 101-103: "A notable symptom related to depression (and is comorbid with other related diagnoses) is the..." is not parallel. Considering the phrase after the "and" renders the predicate to read "symptom ... is ... is." Perhaps the "and" is supposed to be a "that." Additionally, this sentence mistakenly reads that "a ... symptom ... is the rates," which is non-sensical/a category mistake. The authors appear to be meaning to talk about the rates of symptoms rather than saying that the symptom(s) is/are themselves rates.

Thank-you for the insight. The sentence has been rewritten as follows: “Notable symptoms related to depression (which are comorbid with other related diagnoses) are suicidal ideation and attempts to end one’s own life, which have also seen an increase during the COVID-19 pandemic.”

4. P. 14, lines 114-115: "including, and Single Session Therapy" is ungrammatical. Either the "and" should be deleted or another noun is needed before it.

The word “and” has been deleted.

5. P. 15, line 129: A space is needed after "visit."

A space has been added.

6. P. 17, lines 183-184: The phrase "despite an absence of co-located care" is now unnecessary because the prior framing of the paper in terms of integrative care has been changed. In that setting, it is also confusing. Consider deleting.

The phrase has been deleted.

7. P. 32, line 539: The first sentence of the paragraph is ungrammatical with subject predicate disagreement between "is" and "results."

The word “is” has been changed to “are.”

8. P. 32, line 541: There is a comma splice after "WWIC."

The comma has been removed.

9. P. 35, line 626 et passim: The beginning of the sentence is wordy and awkward: "A specific recommendation that may arise from these findings is for programs and service providers to ..." Consider reducing wordiness.

Thank-you for the insight. The sentence has been reworded as follows: “The findings also suggest that single-session therapy can be an accessible and appealing alternative service modality for many clients.”
